# Research capacity of Australian and New Zealand emergency medicine departments

**DOI:** 10.1186/s12245-020-00275-z

**Published:** 2020-04-15

**Authors:** Katie Walker, Shijie Ian Tan, Daniel Fatovich, Gina Watkins, Melanie Stephenson, Joseph Ting, Richard Whittome, Wei Wang, Jonathan Knott, on behalf of the ACEM Clinical Trials Network, Maggie Bock, Michael Stokes, Stephen MacDonald, Peter Jones, Gerben Keijzers, Tegwen Howell

**Affiliations:** 1 Emergency Department, Cabrini, 183 Wattletree Rd, Malvern, Melbourne, Victoria 3144 Australia; 2 Health Services, Monash University, Melbourne, Victoria 3004 Australia; 3 Faculty of Medicine, Nursing and Health Sciences, Monash University, Building 15, 27 Rainforest Walk, Clayton Campus, Wellington Rd, Clayton, Victoria 3800 Australia; 4 Ng Teng Fong General Hospital, 1 Jurong East Street 21, Singapore, 609606 Singapore; 5 Department of Emergency Medicine, Royal Perth Hospital, GPO Box X2213, Perth, 6001 Western Australia; 6 Centre for Clinical Research in Emergency Medicine, Harry Perkins Institute of Medical Research, University of Western Australia, Perth, 6001 Australia; 7 Emergency Department, Sutherland Hospital, Caringbah, Sydney, NSW 2229 Australia; 8 School of Medicine, University of New South Wales, Sydney, New South Wales 2052 Australia; 9 Emergency Department, Austin Hospital, Heidelberg, Victoria 3084 Australia; 10 Mater Hospital, Raymond Terrace, South Brisbane, Queensland 4101 Australia; 11 Ipswich Hospital, Chelmsford Ave, Ipswich, Queensland 4305 Australia; 12 School of Public Health and Social Work, Queensland University of Technology, 2 George St, Brisbane City, Queensland 4000 Australia; 13 Australasian College for Emergency Medicine, West Melbourne, Victoria 3003 Australia; 14 Cabrini Institute, 154 Wattletree Rd, Malvern, Victoria 3144 Australia; 15 Faculty of Medicine, Nursing and Health Sciences, Monash University, 553 St Kilda Rd, Melbourne, Victoria 3004 Australia; 16 Emergency Department, Royal Melbourne Hospital, 300 Grattan St, Parkville, Victoria 3050 Australia; 17 Melbourne Medical School, University of Melbourne, Grattan Street, Melbourne, Victoria 3010 Australia

**Keywords:** Emergency medicine, Research, Research personnel, Surveys and questionnaires, Health resources, Multicentre trials

## Abstract

**Background:**

Large, multicentre studies are required in emergency medicine to advance clinical care and improve patient outcomes. The Australasian College for Emergency Medicine clinical trials network is available to researchers to assist with facilitating large, multicentre research. However, there is no current information about the research capacity of emergency departments (EDs) in Australia and New Zealand.

**Methods:**

All EDs accredited for emergency medicine training in Australia and New Zealand were eligible to participate. Research leads or ED directors were invited via email and telephone to complete a survey. Data were collected regarding the presence of a research lead; their research experience; available research resources including colleagues, funding, departmental paid research time; publications; and research culture.

**Results:**

One hundred and twelve responses were received on behalf of 122 (84%) sites (10 satellite plus main) from a possible 143 sites with all types of hospitals and regions represented. Research leads were identified at 66 (59%) sites; 32 (29%) had a director of emergency medicine research. A wide range of research was underway. Ninety-six sites (66%) contributed data to multicentre projects. Twenty-one centres (17%) were highly productive with multiple resources (skilled colleagues, funding, staffing), a positive research culture and high-volume output. Sixty to seventy centres (50–58%) had limited resources, experienced an unsupportive research culture and authored manuscripts infrequently. Paid time for research directors was associated with increased research outputs.

**Discussion:**

ACEM sites have the capacity to undertake large multicentre studies with a varied network of sites and researchers. While some sites are well equipped for research, the majority of EDs had minimal research output.

## Background

There are over nine million emergency department attendances per annum in Australia and New Zealand [1–3]. Often, current clinical practice is not based upon robust evidence. As we evaluate treatment options, particularly for critically ill patients, we discover many current established therapies have limited efficacy [4, 5]. Multicentre randomised studies have recently been published on core topics such as fluids in sepsis, bronchiolitis management, latrodectism and pneumothorax recommending fundamental changes to patient management [6–11].

To undertake robust studies that properly answer questions about emergency therapies, large multicentre randomised clinical trials are needed. These enrol large numbers of patients, from diverse clinical settings, into well-designed and funded trials. Many patient conditions are identified sporadically in low numbers across hospital networks that vary in patient population and resourcing. Important trials increasingly require adequate funding and resources, skilled investigators and high-functioning clinical trial networks, capable of large-scale project design and delivery [12, 13].

Internationally there are a range of emergency medicine clinical trial networks participating in research. Some, such as the European Society for Emergency Medicine (EUSUM) research network, facilitate trials, other groups undertake international multicentre studies (e.g. Asia, Australia and New Zealand Dyspnoea in Emergency Department (AANZDEM)). Some networks are well established, particularly in paediatrics (Paediatric Emergency Care Applied Research Network (PECARN); Paediatric Research in Emergency Departments International Collaborative (PREDICT)). Many jurisdictions or subspecialties of emergency medicine are forming new collaborations (e.g. Geriatric Emergency care Applied Research (GEAR) network). Networks can be national (New Zealand Emergency Medicine Network (NZEMN)) or international (Pan-Asian Trauma Outcomes Study (PATOS), clinical research network). In order for any of these networks to produce high-quality answers to research questions, individual sites must have the capacity to collect data.

The Australasian College for Emergency Medicine (ACEM) has had clinical trials group delivering studies, which has recently reformed as a clinical trials network (ACEM CTN) [14]. All 143 Australian emergency departments accredited to train emergency medicine residents (ACEM trainees) must support the research learning objectives in the ACEM training curriculum, but the number of sites actively engaged in research has been unknown.

The goal of this study is to describe current research capacity at ACEM training accredited EDs by reporting current resources, funding, activity and research culture. We describe the skill sets and experience levels of emergency medicine research leads, sector and region personnel resources, available paid research time, funding, research outputs, ACEM research training places and research culture.

## Methods

A survey was undertaken of all ACEM accredited EDs between February and April 2019, with invitations sent by email, with telephone follow-up for non-responders. Ethics approval was obtained from Cabrini Human Research Ethics Committee (01-29-10-18). Informed consent was obtained from all participants.

The survey was developed and piloted internally by the authors, being adapted from the Australian and New Zealand Intensive Care Society (ANZICS) survey, see Additional file 1 [15]. Question structures were a combination of pick-lists, five-point Likert scale items and free-text. Some questions were adaptive to responses to reduce the length of the survey, and many sections were mandated. There was a completeness check prior to survey submission, and participants were able to check their answers at any time prior to submission. Only one survey was allowed per site.

All Australian and New Zealand EDs that were accredited for ACEM training in November 2018 were eligible. Australian and New Zealand emergency medicine is largely funded by state and national governments, with universal health coverage for citizens and no out-of-pocket fees for patients. Emergency physicians are usually salaried employees of the government hospitals, with variable amounts of paid time available for non-patient facing work. In Australia, 7% of patients attend private (non-governmental hospitals) and emergency physicians may be paid per hour or per patient as a salaried employee, without governmental support for non-patient facing work. Most university appointments for physicians are honorary/adjunct (unpaid).

EDs were identified from the ACEM accredited training sites database. ED Directors or their Directors of Emergency Medicine Research were invited to participate. The survey was advertised in the weekly ACEM bulletin email. Eligible emergency physicians were emailed a link to the voluntary survey, and they (or delegate) were asked to complete it within 2 weeks. Phone calls were made to non-respondents. Informed consent was obtained, which included information about the research team, survey purpose, the likely time for completion of the survey and data privacy and protection. Data was collected from February 2019 to April 2019, until a survey was complete, a site opted-out or several calls had been made without obtaining data. Missing data was followed up by phone. The survey was closed (invitees only) and was administered online (Redcap, version 8.8.1, Vanderbilt university, TN, USA). The trained data collector (for phone responses) was IT, MS or GW.

Data collected from the ACEM database were site demographics, locations, training accreditation categories and role delineations. Data were collected about emergency physicians, and ACEM trainees were research titles, skills, qualifications and experience levels and the amount of paid research time allocated. Departmental research interests, publication volume, funding amounts and sources for the preceding 5 years were obtained. Publications were defined as “published in a peer-reviewed Medline-listed research publication including at least one ED member as an author”. Grants were defined as “funded grants (The ED must be a major partner in the grant application e.g. principal investigator or major clinical health partner).” Non-emergency physician research staff information was also sought (types of personnel and paid hours available). Qualitative information regarding site research culture was obtained.

All data from complete surveys were included in the analysis. Incomplete/early-termination survey data was also included in the analysis. Descriptive data was summarised using number (%) or median (interquartile range). Likert scores were grouped into negative (very unimportant, not important, neutral) and positive (somewhat important, very important) and reported as percentages. Free text was analysed by two reviewers (MS, KW) using an inductive approach and open coding for themes and content with examples presented.

## Results

### Participant flow

**Fig. 1:**
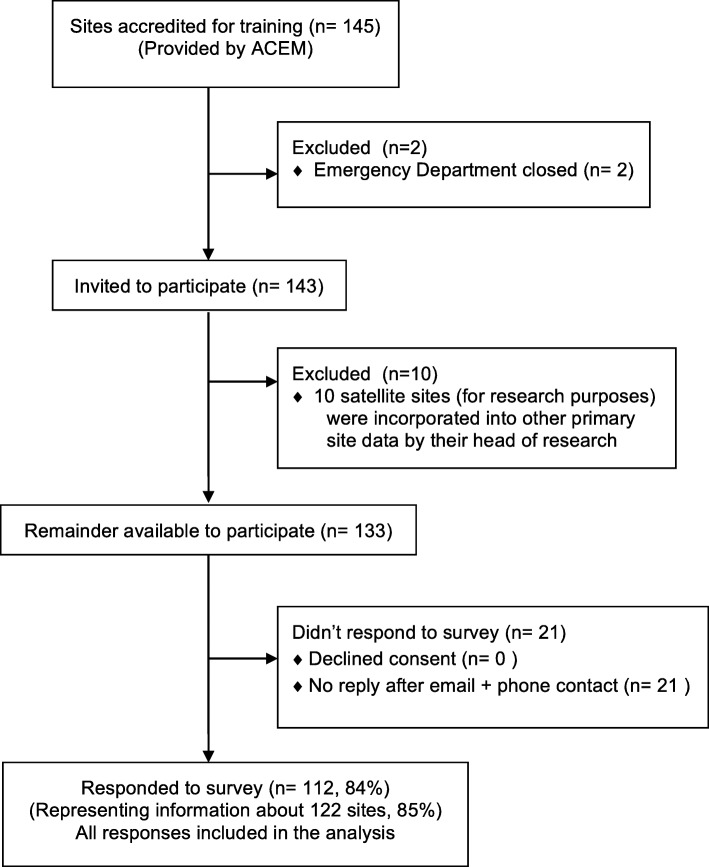
Consort flow diagram for ACEM research capacity study 2019

Study flow proceeded as described in Fig. 1, with 112 survey responses (with 10 people providing combined responses about a main and satellite site: data from 122 physical sites total) from an initial potential 145 sites.

### Participant characteristics

The site characteristics of survey participants are available in Table 1.

**Table 1:** Demographics of sites and participating sites

Characteristics of sites	Total sites number	Responses number (%)
Regions		
Australian Capital Territory	2	1 (50%)
New South Wales	37	26 (70%)
New Zealand	18	13 (72%)
Northern Territory	2	2 (100%)
Queensland	26	24 (92%)
South Australia	7	6 (86%)
Tasmania	2	2 (100%)
Victoria	26	25 (96%)
Western Australia	13	13 (100%)
Aust. Inst. of Health and Welfare, hospital classifications		
(New Zealand)	18	13 (72%)
Private	13	12 (92%)
Large regional	21	20 (95%)
Medium regional	2	2 (100%)
Small/medium regional	6	5 (83%)
Major	30	26 (87%)
Large metropolitan	27	24 (89%)
Medium metropolitan	10	8 (80%)
Specialist children’s	6	2 (33%)
Age of patients		
Adults and paediatric	121	105 (87%)
Adults	5	5 (100%)
Paediatric	7	2 (29%)
ACEM site classifications		
Major referral	38	31 (82%)
Rural/regional base	45	38 (84%)
Urban district	50	43 (86%)
Hospital education accreditation time for each ACEM trainee/resident		
6 months	37	32 (86%)
12 months	35	29 (83%)
18 months	19	12 (63%)
24 months	42	39 (93%)
Total	133	112

Overall, there was an 84% response rate, which was evenly spread across types of hospitals and regions. There was a lower response rate from specialist children’s hospitals, hospitals accredited for 18 months of training and a few states.

**Fig. 2:**
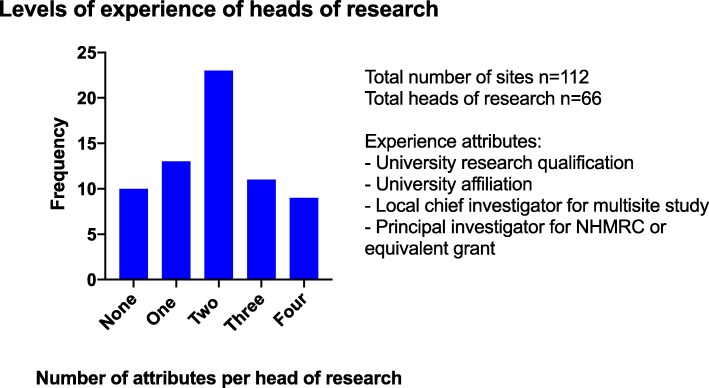
Levels of experience of heads of research

Sixty-six sites (59%) had appointed a head of research, and 32 (29%) held the title of Director of Emergency Medicine Research or similar. All except 2 heads of research were Fellows of the Australasian College for Emergency Medicine (the others both hold a PhD). Amongst the heads of research, there were 12 associate professors and 8 professors. We did not distinguish between adjunct/honorary and full university appointments. Forty-nine (74%) heads of research have a university affiliation; 28 (42%) have been a site chief investigator for a multisite project; 18 (27%) have been a principal investigator on a National Health and Medical Research Council grant. Forty-two university research degrees were awarded to 33 research heads (12 doctoral degrees, 5 doctors of medicine, 13 master’s degrees and 12 other/unknown higher research degrees). The levels of experience of heads of research are shown in Fig. 2.

### Types of research

**Fig. 3:**
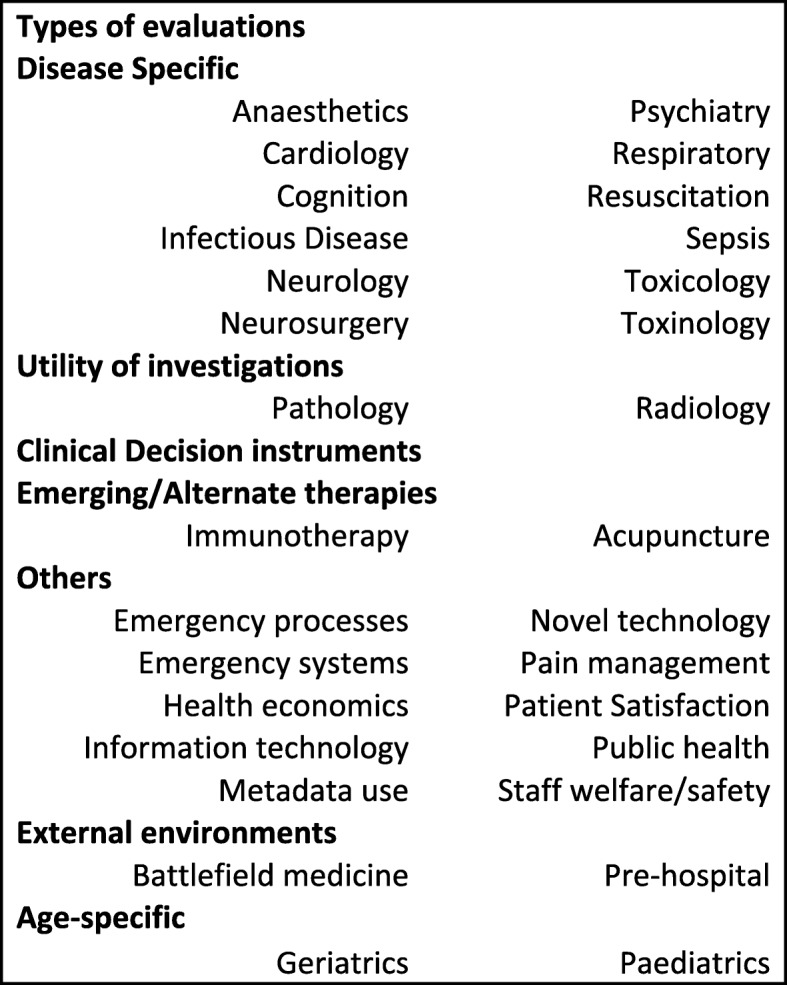
Types of research undertaken at ACEM sites

Departmental research interests varied widely, reflecting the breadth of emergency medicine practice environments and populations (Fig. 3). Ninety-six EDs contributed to multicentre research during the last 5 years. Fifty-two EDs contributed to ACEM CTN projects, mainly collecting data for the ARISE sepsis fluids observational study (*n* = 52) and the spontaneous pneumothorax study (*n* = 19) [11, 16].

### Publications

Overall site contributions to publications were 3336 papers in the last 5 years from the 112 sites. This figure was calculated by summing the number of papers per site but there will have been overlap for multi-site investigations such that the true total number will be lower. Publication volume varied markedly. The 32 sites with a Director of Emergency Medicine Research (or equivalent) appointed published a median of 26.5 papers (*n* = 1814; IQR 8, 67; range 0, 302). The 33 sites with a head of research (excluding those with a Director of Emergency Medicine Research) published a median of 6 papers (*n* = 1237; IQR 1.5, 13; range 0, 502) compared to median of 0 from the 47 sites without a head of research (*n* = 193; IQR 0, 3; range 0, 92). There were 34 sites that did not author a publication in the last 5 years.

### Funding

**Fig. 4:**
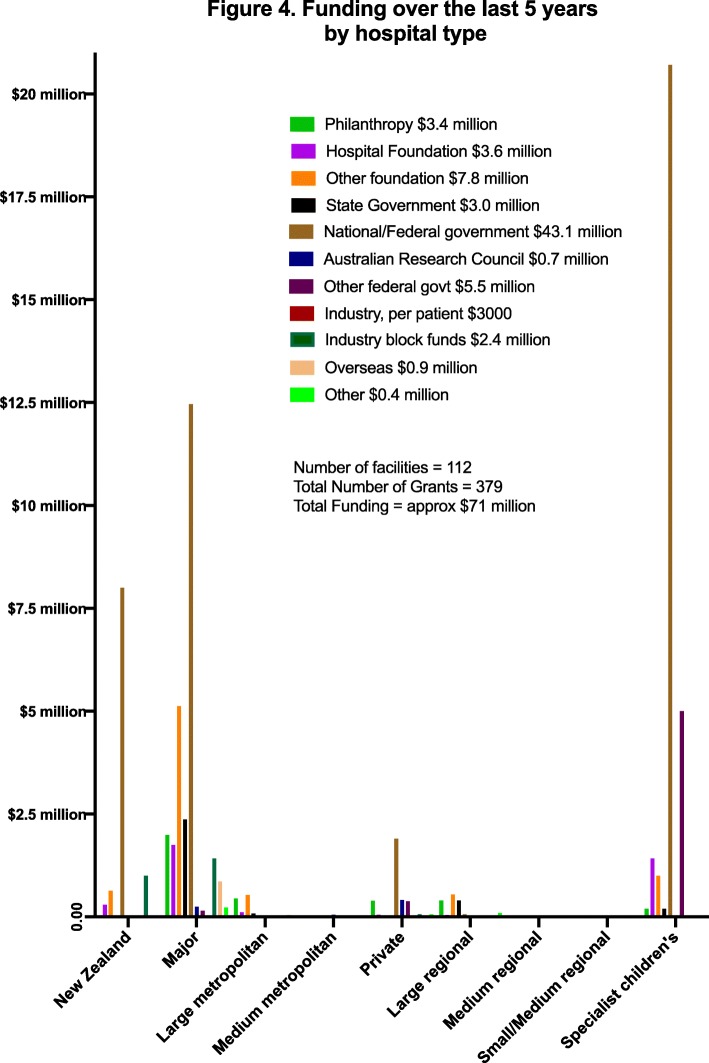
Funding over the last 5 years by hospital type

The total funding achieved over the last 5 years was approximately $71 million AUD (Fig. 4). Of this, $43.1 million was obtained from a national/federal government medical research council, and approximately, $3–7 million each was obtained from philanthropy, hospital foundations, other foundations, state governments, other federal government grants and from block industry grants.

Major metropolitan and specialist children’s hospitals were the most successful in obtaining grants, small to medium hospitals (metropolitan or regional) the least successful. Four regions performed better than others in obtaining funds per head of population (NZ, QLD, VIC, WA) with the other regions obtaining significantly less. The distribution of funding per site was highly skewed.

The median per site value was $0 (IQR $0, $107.5 K; range $0–$21 million). Sixty-six of 112 sites did not achieve any funding and a further 20 achieved less than $150 K. Seven sites obtained over $500 K, 5 over $1 million and the remaining 12 achieved ≥ $1.5 million in grant funding. Sites with a Director of Emergency Medicine Research obtained a median of $105,000 in grants (IQR 5.25, 810 K); sites without a Director of Emergency Medicine Research obtained a median of $0 in grants (head of research IQR 0, 37 K; no research lead IQR 0, 3 K).

### Staffing

Eighty-seven sites had emergency physicians conducting research (median 2, IQR 1, 4). The median fulltime equivalent paid emergency physician research hours were 0.1 (IQR 0, 0.25) or 3.8 h per week. Post-doctoral researchers and other paid researchers worked at 61 sites (median 0, IQR 0, 0.5). Most sites had limited resources in terms of personnel and were reliant on volunteer labour (emergency physicians, other ED staff and 173 medical students). Two sites paid multiple ACEM trainees to conduct research and eight others employed 1–2 trainees.

At the 66 sites with heads of research, the 32 Directors of Emergency Medicine Research were paid a median of 10 research hours per week (IQR 5, 20; range 0, 40). When the head of research didn’t have the Director of Emergency Medicine Research title (33 people) the median paid research time per week was 0 hours per week (IQR 0, 5.5; range 0, 10).

### Culture

Regarding questions about research culture at the EDs, 55% (62/112) thought that emergency medicine research was important to their organisations. Most thought that emergency medicine research was important to their EDs (72%, 81/112). Research leads felt less supported by their fellow emergency physicians with 54% (60/112) giving positive responses and by other ED staff (e.g. nursing co-workers) with 46% (51/112) positive responses. Most felt unsupported when conducting clinical research with a 70% (78/112) negative response rate. Sectors and regions giving more optimistic responses across all questions included specialist children’s, medium regional and New Zealand hospitals. Those struggling included private and medium metropolitan and South Australian hospitals. Free-text responses from 63 respondents identified 3 major and 3 minor themes and examples are presented below.

### Major themes

1. Support
  Some felt well supported: “We have been well supported by the hospital”, “We have good support from colleagues for studies and it is a warm and welcoming environment”. Others were supported in theory without practical support: “Organisation expresses commitment but this is not resourced or matched by reality”. Another group felt completely unsupported: “Deprioritised against service provision”, “no time, support or encouragement locally”.
2. Importance of research at the site
  There were variable thoughts amongst colleagues regarding the importance of research: “It is important for emergency physicians in this ED to be exposed to research projects”, “some (emergency physicians) do not feel clinical research has a role (for them), I have been asked ‘what’s in it for me?’”, “we have very active nursing research”.
3. Research infrastructure as a barrier or enabler
  “Support from the Emergency Medicine Foundation research support network has been invaluable”, “poor infrastructure and support processes create active barriers”. When recruiting patients into clinical trials, some had colleagues who actively supported projects; others declined to recruit patients.

### Minor themes (practical research training, mentoring and advocacy for research)

Some felt that there had been a decline in practical clinical research training since the change to ACEM fellowship training research requirements (allowing course completion instead of manuscript publication or presentation) “we are not developing clinical scientists”, “…created a mindset that there was no benefit to do research” others advocated for mentoring and senior advice for those expressing an interest in clinical research as a career: “…how to set up a research culture….how to actually go about research...” Finally, there was a need identified for advocacy for the importance of research to enable prioritisation of and resource allocation into research, particularly as many felt that there were no resources currently available to them “without resources/funding research isn’t possible”.

## Discussion

This study found an emergency medicine research landscape that is varied. There is research capacity in all types of hospitals and regions with the resources to undertake multi-centre studies. Multiple researchers staff 21 centres (17%), which have excellent infrastructure support, a supportive culture, multiple grants and publications. Sixty to seventy sites rely on volunteers, in-kind donations for infrastructure, experience a lack of funds and have an unsupportive research culture.

This is the first description of the emergency medicine research landscape in Australia and New Zealand. Our findings were similar to the ANZICS research capacity study [15]. Emergency medicine has a higher rate of appointment of a head of research (66% ED, 34% ANZICS), and these researchers have similar levels of skills and experience. A similar proportion of sites were contributing to multicentre studies (86% ED, 80% ANZICS). Emergency medicine had less help from research assistants or coordinators (28% ED, 36% ANZICS).

There is an association between engagement of individuals and organisations with research and improved healthcare performance and patient survival, although this has not been evaluated in emergency medicine [17–19]. Having and paying a Director of Emergency Medicine Research to organise the department’s research was significantly associated with increased departmental research grants and publications. An ED could potentially improve its research contribution by appointing a Director of Emergency Medicine Research and allocating them paid non-clinical support time.

To put emergency medicine research funding into context compared to potential Australian and New Zealand national funding sources, in Australia, $8.8 billion was available to health via the National Health and Medical Research Council (NHMRC) and Medical Research and Futures Fund (MRFF) over the last 5 years and the Australian EDs achieved $35.1 million or 0.4% of these funds (whilst managing 10% of clinical encounters) [20]. Similarly, Australian philanthropic funds were approximately $55 billion, whilst philanthropic funding achieved by Australian EDs was $3.4 million (0.006%) [21].

Many sites expressed interest or were conducting research despite currently feeling that they were being hampered by a lack of resources or governance issues. This group of sites represents untapped potential that with a little support should be able to deliver patient trial data representing their clinical populations.

Departments with a nominated research lead (without funding or allocated research time) undertook more research than those without a nominated lead. An initial step to facilitate multi-site research (without increasing overhead costs) might be to identify a research lead for each site so that researchers initiating multi-site projects are able to invite diverse sites to participate in site data collection.

### Limitations

Retrospective survey methods may introduce non-response, acquiescence, demand characteristic and desirability bias. Paediatric EDs were under-represented. The study focused mainly on emergency medicine research and may (or may not) have under-reported emergency medicine research undertaken by other disciplines such as nursing and allied health groups. Respondents usually only had access to information about themselves and overlapping information would lead to overestimation of grants and publications. There was no attempt made to verify responses for correctness using external sources. Research culture responses may be biased in either direction as researchers and directors provided data. Some regions have centralised research infrastructure and resources, uncaptured by this evaluation.

Site circumstances change over time, and misrepresentations of research lead classifications may have occurred. Many sites recently appointed Directors of Emergency Medicine Research to assist with training accreditation. Some questions relate to the preceding 5 years of research outputs and so it may underestimate the impact of appointing and paying a Director of Emergency Medicine Research.

## Conclusions

ACEM sites have the capacity to undertake large multicentre studies with a varied network of sites and researchers. While some sites are well equipped for research, the majority of EDs had minimal research output.

## Supplementary information

**Additional file 1:.** ACEM Research Capacity Survey

## Data Availability

The datasets generated and analysed during the current study are not publically available to protect participant privacy.
