# Supplementary material for: Research capacity of Australian and New Zealand emergency medicine departments
Source: Int J Emerg Med. 2020 Apr 15;13:16. doi: 10.1186/s12245-020-00275-z (PMC7161130; doi:10.1186/s12245-020-00275-z)
Supplement: Supplementary file 1 — Additional file 1:. ACEM Research Capacity Survey [file 12245_2020_275_MOESM1_ESM.docx]

**ACEM Research Capacity Survey**

Please read the following to decide if you would like to continue and complete the survey.

The Australasian College for Emergency Medicine (ACEM) Clinical Trial Network (CTN) is undertaking a survey of all ACEM training-accredited emergency departments (EDs).

This survey aims to describe the current research capacity of ACEM accredited EDs by describing the current research output and resources of our departments.

This should not take longer than 15 minutes of your time. We appreciate how busy you are and thank you for your help. You may find that you need to look up some of the information required. If you have a record of your departments' recent (5 years: 2014-2018 inclusive) publications, grant sources and approximate grant values, we suggest you open the record now.

If you get interrupted, please save the partially completed survey as the survey will time-out and lose unsaved data if you leave it too long. You can return to it later if you wish. If you have finished the survey, please change the status to "complete" at the end and then save your responses "save and exit". REDCap will provide you with a re-entry code should you need to re-enter data later.

Participation of this survey is voluntary. Your responses will be help the ACEM CTN in planning future multi-centered research as well as helping the new ACEM research committee and potentially other Emergency medicine trial networks such as EMF, PREDICT, EDEN and GEMSIG.

Only one survey should be completed for each Emergency Department and the ED Director, research lead, research coordinator or assistant may undertake this. If you would like to opt out of the survey, please reply via survey question number two with your decision. Your decision will not affect your relationship with ACEM or the ACEM CTN. Please try to reply to the survey within 2 weeks. A reminder will be sent via email if no response is received. A subsequent phone call will be made to request a time to call to complete the survey over the phone if no response is received by 25/2/2019. Missing data will be followed up by phone. If you find that you don't have all the answers immediately to hand, you may re-enter the survey at a later date to add more details. You may choose to opt out of the survey at any time.

Data collected will be used to identify potential regions and sectors in need of support and mentoring as well as the strengths and weaknesses of current trial funding arrangements. Future gains include enabling more of our EDs to have the capacity to support multicentre studies as well as supporting future high-value funding applications.

This study has received Human Research and Ethics Committee approval from Cabrini Hospital. Most data will be

de-identified and only available to the research investigators for the purposes of this project. De-identified data may also be used to support other ongoing emergency medicine research capacity work as well as this study. ACEM will be given a list of the names and email addresses of the research leads at each site and may choose to use this to contact you regarding research issues in the future. If you do not wish this, the opportunity to opt-out of the name/contact sharing is offered to you in the survey.

If there are any questions about the study, ethics approval, the use or storage of data or any other concerns, please feel free to contact Katie Walker to discuss this further. Any complaints regarding the ethical conduct of the trial can be directed to the Cabrini Human Research and Ethics Committee - Manager CHREC [(hrec@cabrini.com.au](mailto:(hrec@cabrini.com.au) or +61 3 9508 3494).

Thank you for your participation. Katie Walker (Cabrini, Vic)

Daniel Fatovich (Centre for Clinical Research in Emergency Medicine, WA)

Ian Tan (Monash University Medical School, Cabrini, Vic) Joseph Ting (Mater Health Services, Qld)

Gina Watkins (Sydney Eye and Ear Hospital, NSW) Jonathan Knott (Royal Melbourne Hospital, Vic)

[kwalker@cabrini.com.au](mailto:kwalker@cabrini.com.au)

+61 431 272 262

Emergency Physician

Director of Emergency Medicine Research, Cabrini, Melbourne Executive Member of ACEM Clinical Trials Network

Adjunct Clinical Associate Professor, Monash University

Name of Hospital

Are you happy to consent to proceed with this survey? Yes

No


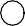

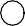


**Department Details**

Director of Emergency Department (Name)

Is the department accredited for specials skills Yes

training in research for ACEM trainees? (Including No provisional accreditation)


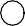

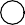


**Research Lead Information**

Does the department have a head of research? Yes No


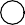

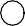


What is their honorific title? Dr

Ms Mr


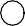

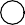

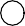

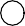

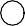

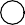


A/Prof Prof Other

Please state their honorific title

First name

Family name

Job title Emergency physician

Director of emergency medicine research Other


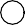

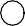

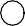


Please state their job title

(e.g. director of emergency medicine)

Email address

We would like to share the research leads' name and Yes email address with ACEM. Do we have permission to do No this? (All other information in the survey will be

de-identified and only made available to researchers)


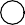

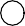


How many paid research hours per week is the head of research allocated?

What is the head of research's ACEM qualifications? FACEM

ACEM trainee Neither

What are the head of research's qualifications and MD by research

levels of experience? (Select all that apply) PhD

Masters of research methodology Masters by research

MPH

Higher research degree (type unknown) Other (higher research degree) University affiliation

A site chief investigator for a multi-centre study since 2014

Chief/principal investigator on an NHMRC or equivalent large grant

None of the above

Please state type of qualification

If there was a thesis subject required for the

research qualification, what was the topic? (Brief description)

University affiliation(s)? Yes

No


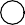

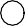


Number of peer-reviewed PubMed MEDLINE listed

publications co-authored by head of research since

January 2014 (Approximate number is fine if exact number is hard to determine.)

**Department Research Funding**

Number of funded grants between 1st January 2014 and

31st December 2018 (The ED must be a major partner in the grant application e.g Principal Investigator

or major clinical health partner)

Approximate value of grants from philanthropic donations

Approximate value of grants from hospital foundations

Approximate value of grants from other foundations

Approximate value of grants from state government

Approximate value of grants from NHMRC

Approximate value of grants from ARC

Approximate value of grants from federal government

(Not NHMRC/ARC)

Approximate value of grants from industry, paid per patient

Approximate value of grants from industry, paid as

block funding

Approximate value of grants from overseas

(Any overseas based source)

Approximate value of grants from others

What was the source?

(We'd appreciate this info if possible, particularly about the sector, fine details not required)

What areas of emergency medicine research is your Hard versus soft collar in cervical spine injury department currently investigating? (Listed are the Outcomes of fluid resuscitation in elderly patients top 10 research priorities as established by the Routine oxygen therapy in acute coronary syndrome clinical trial group in 2014) X-ray before reduction of shoulder dislocation

Ketamine and long-term effects in depression Chest pain protocols

Optimal management of agitated patients Paracetamol vesus celcocoxib in soft tissue injuries

Paracetamol versus placebo in fever

Early versus later steroids in severe sepsis Other

Please state area of emergency medicine research

(Please enter each area on new line.)

Number of peer-reviewed medline-listed research

publications including at least one ED member as an

author since January 2014 (Excluding publications (Approximate number is fine if exact number is counted above in the research lead section) hard to find.)

Has your department participated in multi-centre Yes

research between January 2014 and December 2018? No (Research at ≥3 sites)


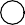

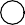


Is the department currently participating in any of No

the ACEM CTN endorsed studies? (Select as many as Fentanyl or placebo with ketamine for RSI (FAKT) applicable) ARISE FLUIDS

ARISE FLUIDS observational study

Identifying patients at risk of post-concussion syndrome after mild TBI

PlasmaLYTE versus Saline to resuscitate patients needing ICU (PLUS)

Conservative versus interventional treatment of spontaneous pneumothorax

Implementing evidence-based recommendations for management of patients with mild traumatic brain injury in Australian EDs

Western Australian Illicit Substance Evaluation

Total number of emergency physicians (FACEMs)

actively undertaking a research project during the

last 12 months in your Department, including the (We are defining research as a project requiring research lead (1st January 2018 to 31st December ethics approval, whose results will be published 2018) in a medline-listed peer-reviewed journal, not

including audits or quality improvement projects)

Average utilised FTE of paid, non-clinical FACEM time

for conducting research (1st January 2018 to 31st December 2018) (Please include the research lead)

Total number of other researchers in department (i.e.

scientists or other suitably qualified individuals engaged in significant research activity on January

1st 2019)

Total number of other researcher coordinators,

research nurses, lab assistants, research assistants and trial managers involved in emergency medicine

research on January 1st 2019

Total research FTE of other researcher coordinators,

research nurses, lab assistants, research assistants and trial managers involved in emergency medicine

research on January 1st 2019

Total unpaid research students (i.e. medical

students, junior doctors, ACEM trainees, others) involved in emergency medicine research on January

1st 2019

How many ACEM trainees had paid clinical support time

to undertake a research project on January 1st 2019?

**We would like to understand the skill sets and research experience of our ACEM researchers.**

**Please could you answer the following for each team member (Do exclude the research lead whose details have been filled above)**

What is the name of your ACEM trainee or fellow

undertaking research on January 1st 2019? (Exclude

research lead details) (Please leave empty if you have finished listing your trainees or fellows)

What is his or her ACEM qualification? FACEM

ACEM trainee

(Please leave unselected/unchecked if you have finished listing your trainees or fellows)

What are his or her qualifications and levels of MD by research

experience? (Select all that apply) PhD

Masters of research methodology Masters by research

MPH

Higher research degree (type unknown) Other (higher research degree) University affiliation

A site chief investigator for a multi-centre study since 2014

Chief/principal investigator on an NHMRC or equivalent large grant

None of the above

(Please leave unselected/unchecked if you have finished listing your trainees or fellows)

**Perceptions of Research Culture**

How important do you feel emergency medicine research is to your organisation?

Very important Somewhat

important


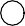

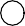


Neutral Not important Very

unimportant


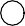

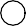

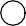


Comments

How important do you feel emergency medicine research is to this ED?

Very important Somewhat

important


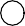

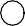


Neutral Not important Very

unimportant


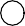

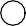

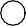


Comments

How important do you feel emergency medicine research is to this ED's FACEMs?

Very important Somewhat

important


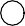

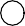


Neutral Not important Very

unimportant


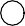

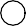

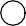


Comments

How important do you feel emergency medicine research is to other ED staff (e.g nurses, junior doctors)?

Very important Somewhat

important


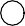

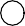


Neutral Not important Very

unimportant


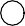

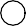

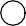


Comments

How well supported do you feel when you conduct clinical emergency medicine research?

Extremely

well supported


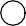


Well

supported


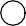


Neutral Not well

supported


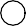

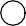


No support at

all


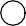


Not applicable


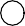


Comments


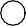
How well supported do you feel by your ED colleagues when you need them to recruit patients into clinical trials?

Extremely well supported

Well supported


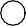


Neutral Not well supported


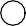

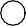


No support at all


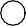


Not applicable


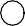


Comments

**Final Comments**

Any final comments about research/research capacity/emergency medicine funding/this study?

**Your Details**

Name and Title

Phone number

Email address
